# Fungal Pathogens in Pet Dogs and Cats in Grenada: Identification and Antifungal Susceptibility

**DOI:** 10.3390/jof11080590

**Published:** 2025-08-12

**Authors:** Erica Hazel-Ann Brathwaite, Kamashi Kumar, Grace Dolphin-Bond, Wayne Sylvester, Victor Amadi, Andy Alhassan

**Affiliations:** 1 School of Veterinary Medicine, St. George’s University, True Blue, St. George’s, Grenada; ebrathwaite@sgu.edu (E.H.-A.B.); kakumar@sgu.edu (K.K.); wsylvester@sgu.edu (W.S.); 2 School of Medicine, St. George’s University, True Blue, St. George’s, Grenada; dolgra@sgu.edu; 3 Dallas County Health and Human Service, Stemmons, TX 75207, USA; vamadi30@gmail.com; 4 School of Public Health and Preventive Medicine, St. George’s University, True Blue, St. George’s, Grenada

**Keywords:** prevalence, dermatophytes, zoophilic, yeast, commensal, susceptibility, fungal

## Abstract

Considering the clinical relevance of commensal yeasts (*Malassezia* and *Candida*) and zoophilic dermatophytes (*Microsporum canis* and *Trichophyton mentagrophytes*) in dogs and cats, this study determines the prevalence of fungal species involved in ear and superficial skin infections in dogs and cats in Grenada and examines their antifungal susceptibility. The etiological agents were isolated from ear, skin, and hair samples of suspected clinical fungal cases using Sabouraud Dextrose Agar (SAB). The isolates’ identification comprised morphological, biochemical, and molecular methods encompassing micro-/macroscopy analysis. Biochemically, yeast isolates were identified by the BD Phoenix M50 microbial identification system, and additional validation of all fungal isolates was performed by polymerase chain reaction (PCR) and sequencing of the ITS region. Furthermore, the E-Test (Epsilometer Test) was used to determine the susceptibility patterns for four azole drugs: ketoconazole, itraconazole, fluconazole, and voriconazole. A total of 405 samples (266 ear, 61 skin, and 78 hair) were collected from 136 dogs and 43 cats. The identified species were *Malassezia pachydermatis*, *Candida tropicalis*, and *Trichophyton* spp. All isolates demonstrated (100%) resistant activity to fluconazole. Importantly, this knowledge will significantly contribute to our understanding of the epidemiology of fungal infections as well as provide guidelines for preventive measures against fungal infections in Grenada.

## 1. Introduction

Fungal pathogens represent a growing concern in veterinary medicine, particularly in tropical regions where environmental conditions favor fungal proliferation. In dogs and cats, superficial mycoses are commonly caused by dermatophytes, such as *Microsporum* spp. and *Trichophyton* spp., and by yeasts, notably *Malassezia pachydermatis* and various *Candida* species [1]. These pathogens are responsible for conditions including dermatophytosis (ringworm), *Malassezia*-induced dermatitis, and candidiasis, affecting keratinized tissues such as skin, nails, and hair [1,2]. Fungal infections in companion animals are influenced by various environmental and physiological factors, including climate, immune status, and fungal spores in the environment. Their prevalence is notably higher in tropical and subtropical climates, where warm and humid conditions favor fungal proliferation [3]. Other predisposing factors include age (<1 year), indoor vs. outdoor pets, adherence to moisture on skin, and nutritional status [2]. Globally, the incidence of these infections has increased and is driven by rising exposure, immunosuppression, and environmental contamination [1,4].

Dermatophytes are of particular concern because of their zoonotic potential. Zoophilic species, such as *Microsporum canis* and *Trichophyton mentagrophytes*, are commonly isolated in pet dogs and cats. *M. canis* is most frequently found in cats, while *T. mentagrophytes* predominates in dogs [1,2,5]. Epidemiological studies report dermatophyte prevalence rates ranging from 13% to 38.9% in dogs and cats, respectively, with higher rates observed in cats [2,3]. In humans, zoophilic dermatophytes usually cause infection of the skin, nails, and scalp. Transmission typically occurs through direct contact with infected animals or contaminated environments, posing a significant public health risk, especially to immunocompromised individuals, but its significance lies particularly in households where pets coexist with humans [6].

Yeast infections caused by *Malassezia* and *Candida* species are also common in companion animals. *Malassezia pachydermatis* is frequently associated with otitis externa and dermatitis in dogs, and to a lesser extent in cats [1,7], and is 80% prevalent in the skin and ears of dogs. Although typically commensal, *Candida* is less than 20% prevalent [4] to cause diseases in dogs and cats. However, *Candida* species can cause opportunistic infections in mucocutaneous areas, particularly under conditions of immune suppression [5,8]. Furthermore, *Malassezia pachydermatis* has some zoonotic potential and has been implicated in nosocomial infections in low-birth-weight human neonates [3,7], while *Candida* species generally pose a lower zoonotic risk. The virulence of these organisms depends on the health status of the host animal [9].

Antifungal therapy is categorized into four main groups: azoles, allylamines, polyenes, and echinocandins. Azoles are commonly recommended for both human and veterinary use [10]. However, antifungal resistance is a growing concern, exacerbated by improper or excessive antifungal use [11]. While many yeast strains (*C. glabrata, M. pachydermatis*) remain susceptible to azoles [12,13], resistance, particularly among some *Candida* species, is increasingly reported [7]. According to the World Health Organization [14], azole resistance in *Candida* species has become a global concern. This resistance has been linked to mutations such as A395T in the ERG11 gene (encodes the enzyme lanosterol 14α-demethylase, which is involved in the biosynthesis of ergosterol, an essential component of fungal cell membranes) and the overexpression of ERG11 and UPC2 (involved in regulating sterol biosynthesis in yeast which plays a crucial role in azole antifungal resistance) [15].

Despite frequent clinical suspicion of fungal infections in pets, there is limited knowledge regarding fungal diseases in Grenada, and the overall prevalence of these diseases remains unestablished. Furthermore, no published studies have investigated fungal diseases in pet dogs and cats in Grenada or elsewhere in the Caribbean, except a study on dermatophytes in stray dogs and cats in Puerto Rico [16]. This knowledge gap is particularly concerning given the growing threat of fungal diseases to animal health and the limited data on fungal species and antifungal susceptibility patterns in Grenada. Given the favorable environmental conditions for fungal proliferation, the zoonotic risks, and the rise in antifungal resistance, there is an urgent need to investigate fungal pathogens affecting Grenada’s pet population and to evaluate their susceptibility to commonly used antifungal agents and the public health implications on the island.

## 2. Materials and Methods

### 2.1. Sample Collection, Processing, and Isolation

Four hundred and five (405) samples of suspected fungal infection were examined and cultured from pet dogs and cats. The samples were collected from the different parishes in Grenada by a veterinarian and sent immediately to the St. George’s University (SGU) Bacteriology and Mycology laboratory for processing. Commercial sterile cotton swabs, containing Amies agar gel transport medium, were used to collect the samples from the ear and skin. Hair samples were plucked from suspected superficial skin lesions and placed into clean Ziplock bags. The samples were cultured onto SAB (Oxid, USA) and incubated at 25–37 °C for up to 3 days for isolation of *Candida* spp., up to 5 days for *Malassezia* spp., and up to 14–21 days for dermatophyte spp. The collected samples were stored for up to one week at 4 °C. Isolates were stored on SAB for yeast and dermatophyte test medium (DTM) for molds at −80 °C for further analysis.

### 2.2. Morphological and Biochemical Identification

The isolates were morphologically categorized using traditional methods [8]. Lactophenol cotton blue (LPCB) stain was used to stain suspected dermatophyte colonies (Volu-sol, Fisher Scientific USA). *Microsporum* spp. are spindled or boat-shaped, and *Trichophyton* spp. are mostly cigar-shaped. The morphologic features of *Candida* spp. are oval to round budding yeast cells, stained blue, and *Malassezia* are peanut footprint-shaped yeast cells. Biochemically, the BD Phoenix M50 identification machine (Decton, Dickin and Co., NJ, USA) was used to identify and confirm isolated yeast species according to the manufacturer’s instructions and protocols. A 2.0–2.4 McFarland suspension (3–5 colonies) in the ID broth was prepared from a 24–48 h culture and vortexed for about 1 min. The turbidity standard was measured using a nephelometer (Decton, Dickin and Co., NJ, USA). The suspension was poured into ID 51 wells, covered, and scanned into the M50 machine. This ID process took 16–24 h, after which the automated system generated the ID results.

### 2.3. Molecular Confirmation of Isolates

DNA Extraction: All fungal strains were re-cultured on Sabouraud Dextrose Agar and incubated at 25 °C for 4–7 days. For DNA extraction, mycelia and spores were collected and placed in 1.5 mL of 1× Phosphate-Buffered Saline (PBS) according to the following protocols [17]. Approximately 15 to 20 silica glass beads (1.0 mm) were added to 200 µL of the stored samples and beaten at high speed for 1 min, with a 30 s interval, using a BioSpec1001 mini-Bead beater (BioSpec Products Inc., USA). Fungal DNA was extracted using the Qiagen micro-mini kit and the Qiagen quick-start protocol (Qiagen, MD, USA). A Nanodrop 2000 spectrophotometer (Thermo Fisher Scientific, USA) was used to measure the concentration and purity of the DNA. The extracted DNA was stored at minus 20 °C (Thermo Scientific, USA) until amplification. PCR Primers: The Internal Transcribed Spacer (ITS) 1 and 4 general fungal primer sets were used to target the rDNA regions of all isolates (ITS1-5′TCCGTAGGTGAACCTGCCG-′3), (ITS4-5′-TCCGCTTATTGATATGC-′3) [6,9]. In addition, yeast isolates were further confirmed using NL1-F(5′GCATATCAATAAGCGGAGGA-′3) and NL4-R (5-′TTGGTCCGTGTTTCAAGACG′-3), targeting the D1-D2 region [18]. Dermatophyte isolates, especially Trichophyton species, were further confirmed using primer pairs LR1-F (5′-GGTTGGTTTCTTTTCCT′-3) and SR6R (5′-AAGTAAAAGTCGTAACAAGG′-3) [6,19]. Amplification: For PCR amplification, 2 µL of DNA (40 µg/mL) from each of the isolates was mixed with PCR mixture Dream Taq Green Master Mix 2× (Thermo Scientific, USA) carried out in a 25 µL final reaction volume for all primer pairs (ITS 1/ITS 4, NL-1/NL-4, LR1/SR6) using the Gene Amp PCR System (Applied BioSystems, CA, USA) thermocycler. According to the manufacturer’s protocols, the reaction mixture contained 12.5 µL of Dream Taq, 7.5 µL of molecular-grade water, and 1.5 µL of forward and reverse primers. Nuclease-free water was used as a negative control in all reactions, and *Candida albicans* ATCC 2091 was used as the positive control for the ITS and NL primer reactions. *Trichophyton mentagrophytes* ATCC 9533 was used as the positive control for dermatophyte reactions with LR1 and SR6 primers. Cycling protocols: Each general primer reaction consisted of initial preheating to 95 °C for 5 min, followed by 35 cycles of 1 min at 95 °C, 1 min at 53 °C, 1 min at 72 °C, and a final extension at 72 °C for 7 min and holding at 4 °C. The reaction protocols for NL1 and NL4 mixture were as follows: denaturation at 94 °C for 3 min, then 40 cycles of denaturation at 94 °C for 1 min, annealing at 53 °C for 1 min, and extension at 72 °C for 1 min, followed by a final extension at 72 °C for 5 min and holding at 4 °C [18]. The LR1 and SR6 reaction protocols consisted of initial preheating to 95 °C for 7 min, followed by 35 cycles of 1 min at 94 °C 40 s, 1 min at 56 °C, 45 s at 72 °C for 1 min, and a final extension at 72 °C for 7 min and holding at 4 °C [20]. The PCR products for each target amplicon were electrophoresed on a 1.5% agarose gel (TopVison Agarose tablet, Thermo Scientific) in 100 mL of Tris-acetate-EDTA (TAE) 1× buffer (40 mM Tris, 20 mM Acetate, 1 mM EDTA, and pH 8.6). DNA bands were detected using the Enduro GDS (Labnet International Inc., Edison, NJ, USA) gel imaging system. DNA Sequencing: PCR-positive amplicons of the expected size were extracted using the Qiagen QIAquick Gel Extraction Kit according to the manufacturer’s directions. Cleaned amplicons (PCR products) were sequenced by Molecular Cloning Laboratories (MCLAB) (San Francisco, CA, USA) using the ITS primers. Representative isolates of *Malassezia pachydermatis* (Mp-GND1), *Candida tropicalis* (Ct-GND1), and *Trichophyton mentagrophytes* (Tm-GND1) were submitted to GenBank at the National Center for Biotechnology Information (NCBI) under the accession numbers PV839438, PV837987, and PV837986, respectively. Phylogenetic tree. The *T. mentagrophytes* isolate Tm-GND1 obtained in this study was used to construct a phylogenetic tree based on the internal transcribed spacer (ITS) region. Multiple sequence alignment was performed using MEGA version 12 (https://www.megasoftware.net, accessed on 23 June 2025), with reference sequences retrieved from the NCBI GenBank database for comparative analysis [21].

### 2.4. Antifungal Susceptibility Testing

The antifungal susceptibility profile of the fungal isolates followed the Clinical Laboratory Standard Institute (CLSI) M44A standard protocol [22]. The four antifungal drugs tested were Ketoconazole (KE) 0.002–32 µg/mL, Voriconazole 0.0002–32 µg/mL, Itraconazole 0.002–32 µg/mL, and Fluconazole 0.016–256 µg/mL using MIC test strips (Liofilchem, Roseto degli Abruzzi TE, Italy). MIC strips were stored at −20 °C until the tests were performed. A homogenous mixture of the fungal isolates was prepared using the McFarland 2.0–2.4 turbidity standard. Mueller Hinton agar plates containing 2% dextrose were incubated at 25–37 °C for 24–48 h, and the minimum inhibitory concentration (MIC) values were visually assessed and recorded. The MIC values were read according to the CLSI clinical breakpoints for each species.

### 2.5. Data Analysis

Statistical analyses were accomplished utilizing R software (https://www.r-project.org/, accessed on 23 June 2025). A Fisher’s Exact test was used to determine the prevalence rate of dermatophytosis and yeast infection in dogs and cats. *p*-values of <0.05 were considered significant.

## 3. Results

### 3.1. Morphological and Biochemical Identification

In this study, 405 samples were examined and cultured from 136 dogs and 43 cats, of which 266 were ear, 61 were skin, and 78 were hair. Twenty-four (*n* = 24) pets were <1 year, fifty-four (*n* = 54) were 1–5 years, and fifty-eight (*n* = 58) were >5 years; forty-three (*n* = 43) of the pets’ ages were unknown (Table 1). A total of 79 fungal isolates belonging to the genera *Trichophyton* (*n* = 2), *Malassezia* (*n* = 56), and *Candida* (*n* = 21) were recovered. Based on the macroscopic and microscopic examination (Figure S1), the two dermatophyte isolates were identified as belonging to the Trichophyton genus, recovered from 2.5% of the hair samples cultured. Biochemically, the yeast isolates were identified as *M. pachydermatis* and *C. tropicalis*, recovered from the ear (21%) and skin swabs (3.2%). This result showed that *M. pachydermatis* was 23% (*n* = 54) isolated from dogs’ ears and 6.25% (*n* = 2) isolated from cats’ ears. *C. tropicalis* was 7.6% (*n* = 21) isolated from skin and ear samples of dogs only (Table 2).

### 3.2. Molecular Confirmation of Isolates

**Figure 1:**
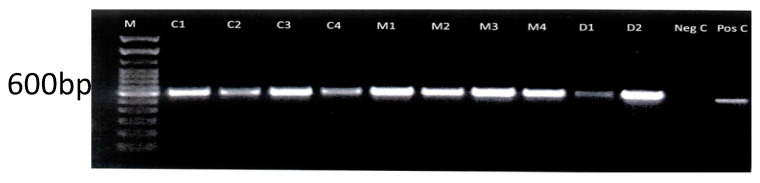
Agarose gel electrophoresis of selected PCR products of fungi isolates with ITS-1 and ITS-4 general fungi primers. Lane M indicates a 100 bp DNA ladder. Lanes C1–C4 indicate *C. tropicalis*. Lanes M5–M8 indicate *M. pachydermatis.* Lanes D1–D2 indicate *Trichophyton* spp. Neg C = negative control (water); no band is present. Pos C = positive control; *C. albicans* ATCC2091 (600 bp).

**Figure 2:**
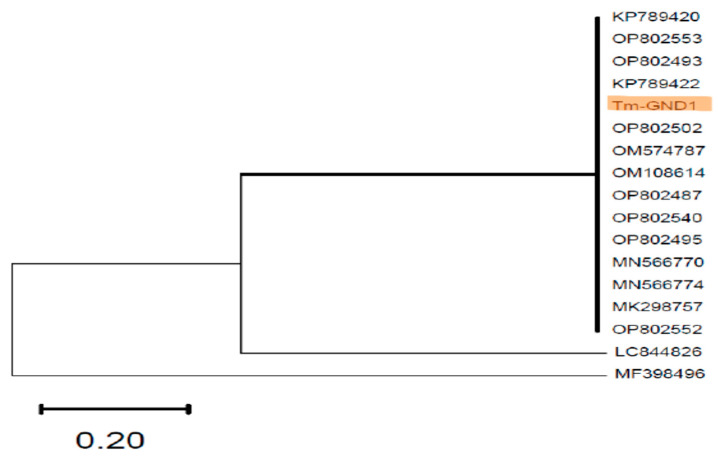
Phylogenetic tree of the Grenadian *Trichophyton* isolate (shaded) based on the ITS region. The phylogenetic tree was inferred using the Maximum Likelihood method based on the Tamura–Nei **(TN94)** Model, with 1000 bootstrap replicates.

All identified fungi were confirmed by PCR using universal ITS-1 and ITS-4 primers, amplifying the 5.8 S region of the ribosomal cistron at 600 bp. The amplicons observed for selected fungi isolates using ITS-1/ITS-2 primers are illustrated in Figure 1. The fungi identified as yeast were confirmed by NL-1 and NL-2 primers specific for targeting the D1D2 region of the large ribosomal subunit, which has notable discrimination for yeast with PCR bands at approximately 620 bp. Dermatophyte isolates identified as Trichophyton were confirmed by PCR using primers LR1 and SR6 specifically for the amplification of the ITS 5.8 S ribosomal cistron of dermatophytes, especially Trichophyton, at 630–800 bp. Based on the sequence analysis of the ITS region of the Grenadian dermatophyte isolate, a phylogenetic tree was constructed. The partially sequenced region showed similarity to other dermatophyte species in the Gene Bank. Of the isolates, ITS genotypes align with specific known dermatophyte strains with similar ITS sequences, especially trichophyton species (Figure 2).

### 3.3. Antifungal Susceptibility Results (AFST)

Table 3 presents the in vitro susceptibility profile of four azole drugs commonly used in Grenada, either topically or orally (Figure S2). Resistance activity was observed in all isolates to fluconazole (100%). The full antibiogram is listed in Table 3 and Table S1.

## 4. Discussion

Fungal species responsible for skin and ear infections in the pet population of Grenada are not well documented, and the true prevalence of such infections remains unknown. Despite the growing threat that fungal diseases pose to animal health, there is limited knowledge regarding the specific fungal species involved and their antifungal susceptibility patterns. This study analyzed clinical samples from pet dogs and cats to assess the current status of fungal infections in Grenada. According to published studies [23], dermatophytosis is more commonly observed in younger pets (<1 year of age); however, no significant differences were found in the prevalence of dermatophyte infection in pet dogs and cats, attributed to the age predisposing factor for dermatophytosis, with a *p*-value of 0.05. The sampled dogs and cats of that age were negative. Additionally, the prevalence of dermatophytosis in cats was insignificant. Factors such as hair length and environmental exposure can significantly influence dermatophytosis in cats [24]. This may influence the absence of dermatophyte growth in the sampled cat population, most of which was indoor domestic short-haired cats. The relatively low rates of dermatophytosis observed in the pets on the island may be attributed to several factors. These include the likelihood that household or family pets receive timely veterinary care, differences in pet age demographics, the ratio of stray to indoor pets, and specific environmental conditions. Pets belonging to Grenadian households and those owned by university students often benefit from the One Health One Medicine initiative led by the School of Veterinary Medicine at St. George’s University. This initiative may have significantly contributed to early diagnosis and treatment, potentially explaining the lower observed infection rates. Currently, no published data exist on dermatophyte prevalence in Caribbean pets, but studies conducted in countries with comparable climates, such as Brazil, Thailand, and Turkey, have reported high prevalence rates [2,3,24]. The most commonly isolated dermatophyte in this study was *Trichophyton* spp., found solely in dogs; this aligns with previous reports indicating that *Trichophyton species* are more commonly isolated from dogs than cats [2,3]. The isolation of *Trichophyton* in Grenadian dogs is significant because of its zoonotic potential and the associated risk of human infection. The spores of this fungus can remain viable on pet bedding, shed hairs, and skin particles for 6–12 months [2]. While the sampled cats were negative for any dermatophyte, it is important to note that dermatophytosis is more predominant in cats than dogs, with *Microsporum canis* being the most common etiological pathogen. The risk of infection is compounded by exposure to vectors and contaminated and crowded environments [25]. However, the prevalence varies geographically because of alterations in weather patterns [3].

In addition to dermatophytes, *Malassezia pachydermatis* and *Candida tropicalis* were also isolated. *M. pachydermatis* was the most frequently identified yeast, found in 90% of the ear swab samples, primarily from dogs. This supports earlier studies by [4,25], which reported higher colonization rates in dogs’ ears compared to those of cats. While not typically part of the normal human skin microbiota, *M. pachydermatis* has been implicated in opportunistic infections, especially among immunocompromised individuals and low-birth-weight infants in neonatal intensive care units [4,26]. Furthermore, *Candida tropicalis* was the only *Candida* species isolated in this study, identified in both the ear and skin samples of dogs. Although *C. tropicalis* is a common endogenous saprophyte, its pathogenic role is well recognized, and it has been frequently isolated from otherwise healthy animals [4]

Treatment of mycotic infections in Grenadian pets typically involves the use of azole antifungals, including ketoconazole, voriconazole, itraconazole, and fluconazole. These agents are commonly used in both human and veterinary medicine, which has contributed to the development of antifungal resistance, particularly to fluconazole [7,11,27]. Antifungal susceptibility testing in this study revealed that *Malassezia* isolates were highly susceptible to voriconazole and ketoconazole. However, one of the most significant findings was the resistance of all the tested yeast isolates to fluconazole. This observation is consistent with other studies reporting high levels of azole resistance in fungal isolates from animals, particularly resistance to fluconazole [28]. While the factors contributing to this resistant activity are not yet known on the island, this can likely be a consequence of the frequent misuse or overuse of fluconazole. Other contributing factors include the widespread use of azoles in agriculture and animal feed [29]. In addition, other important mechanisms driving resistance include overexpression of resistance genes, such as ERG11 and ERG5, chromosomal alterations, and changes in sterol import pathways [27,29,30]. Nevertheless, the actual factors of antifungal resistance in Grenada’s pet population remain unclear and merit further investigation.

## 5. Conclusions

The findings of this study highlight the significance of fungal infections in Grenada, particularly those affecting the canine population, with *Trichophyton* species identified as the primary agents of infection, while *Microsporum canis* was absent. This absence may be due to factors such as the predominantly indoor lifestyle of cats, limited environmental exposure, and access to adequate veterinary care. Given the close contact between humans and their companion animals, especially dogs and cats, preventative measures should be taken to prevent zoonotic transmission. *Microsporum pachydermatis* was the most frequently isolated fungus and the most etiological agent from the ear samples. Additionally, this study provides valuable insights into the antifungal susceptibility patterns among fungal isolates from dogs and cats in Grenada. The yeast isolates demonstrated 100% resistance to fluconazole.

## Figures and Tables

**Table 1:** Profiles of dogs and cats with suspected cases of fungal diseases.

Animal				
		Dog (*n* = 136)	Cat (*n* = 43)	Total (*n* = 179)
Age	<1 year old 1–5 years old >5 years old unknown	15 (11%) 35 (25.7%) 47 (34.5%) 39 (28.6%)	9 (20.9%) 19 (44.1%) 11 (25.5%) 4 (9.3%)	24 (13.4%) 54 (30.1%) 58 (32.4%) 43 (24%)
Sex	Female Male Unknown	77 (56.6%) 46 (33.8%) 13 (9.5%)	18 ((41.8%) 25 (58.1) 0 (0%)	95 (53%) 71 (39.65) 13 (7.2%)
Breed	Pot hound Mixed German shepherd Pitt bull Rottweiler Labrador Others Domestic short hair Siamese	54 (39.7%) 55 (40.4%) 5 (3.6%) 6 (4.4%) 4 (2.9%) 7 (5.1%) 12 (8.8)	42 (97.7%) 1 (2.3%)	

Total number of dogs and cats sampled. Most dogs and cats were >5 years old. Most cats were domestic short-breed.

**Table 2:** Epidemiological numbers.

Variable	Categories	# Of Subjects	% Positive *M. pachydermatis*	% Positive *C. tropicalis*	% Positive Dermatophytes
**Age**	Young (<1 Year)	24	16.6%	4.1%	0%
	Adult (>1 Year)	97	18.5%	7.2%	2%
**Species**	Dog	136	14.7%	6.6%	1.4%
	Cat	43	2.3%	0%	0%
**Ear samples**	Dog	234	11.5%	3.4%	*
	Cat	32	6.2%	0%	*
**Skin samples**	Dog	44	0%	4.5%	*
	Cat	17	0%	0%	*
**Hair samples**	Dog	57	*	*	3.6%
	Cat	21	*	*	0%
***p*** **-value**			0.19	0.14	0.67

Prevalence of dermatophytes and yeast (*Malassezia* and *Candida*) in relation to the data in a population of 136 pet dogs and 43 pet cats. Statistical significance was set at *p* < 0.05. * not applicable.

**Table 3:** Antifungal susceptibility profile of the 79 isolates.

Antifungal Drug	*Malassezia* (*n* = 56)				*Candida* (*n* = 21)				Dermatophyte (*n* = 2)			
	#S	#I	#R	%R	#S	#I	#R	%R	#S	#I	#R	%R
**Voriconazole**	54	0	0	0%	2	19	0	0%	0		2	100%
**Itraconazole**	42	*	14	25%	10	*	11	52%	2		0	0%
**Fluconazole**	0	0	56	100%	0	0	21	100%	*	*	2	100%
**Ketoconazole**	56	*	0	0%	0	*	21	100%	*	*	*	

Note. *n*: number, %: (percentage), S = sensitive. I = intermediate. R = resistant, * No CLSI set breakpoints.

## Data Availability

The original contributions presented in this study are included in the article/Supplementary Material. Further inquiries can be directed to the corresponding author.

## Supplementary Materials

The following supporting information can be downloaded at: https://www.mdpi.com/article/10.3390/jof11080590/s1, Figure S1. Macroscopic and microspic isolates of *Trichophyton* spp. Figure S2. Antimicrobial suceptibility test. Mueller Hinton agar /w 5% dextrose containing Epsilometer test (E-Test) strips of voriconazole, itraconazole, fluconazole, and ketoconazole. Table S1: MIC values of 4 azole drugs against 79 clinical fungi isolates from pet dogs and cats.
