# Supplementary material for: Fungal Pathogens in Pet Dogs and Cats in Grenada: Identification and Antifungal Susceptibility
Source: J Fungi (Basel). 2025 Aug 12;11(8):590. doi: 10.3390/jof11080590 (PMC12387588; doi:10.3390/jof11080590)
Supplement: Supplementary file 1 [file jof-11-00590-s001.zip › jof-3670210-supplementary.pdf]

Figure S1. Macroscopic and microspic isolates of *Trichophyton spp*

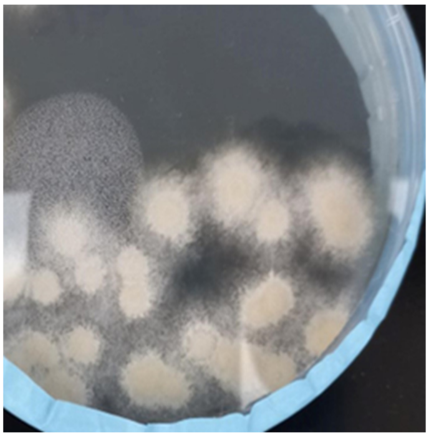

S1A. *Trichophyton spp* -SAB

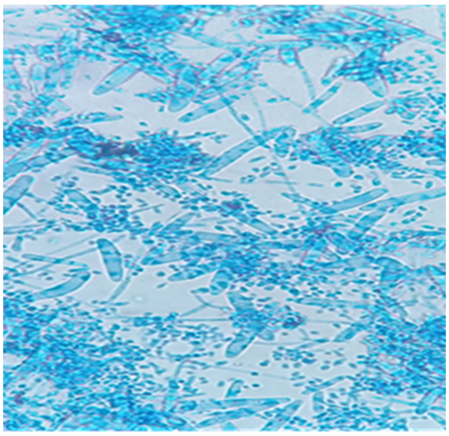

S1A. *Trichophyton spp* -Gram stain

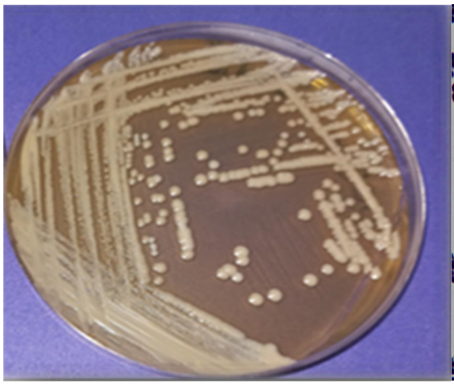

S1B. *Candida tropicalis*-SAB

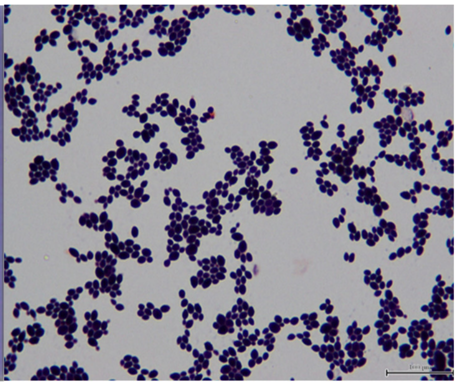

S1B. *Candida tropicalis* – Gram Stain

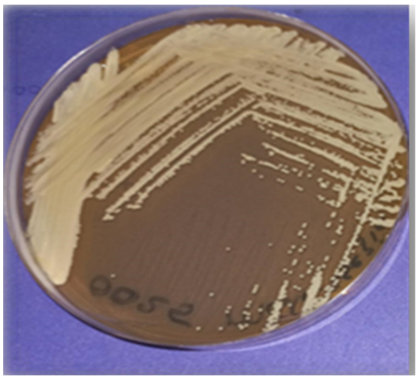

S1C *M. pachydermatis* – SAB

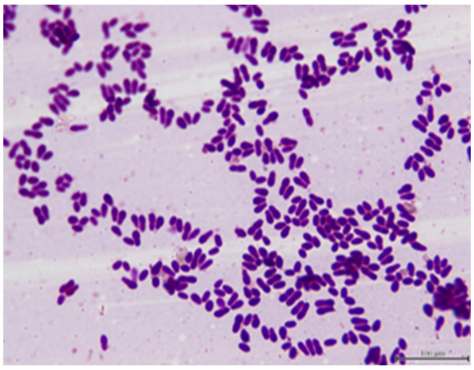

S1C. *M. pachydermatis* – Gram Stain

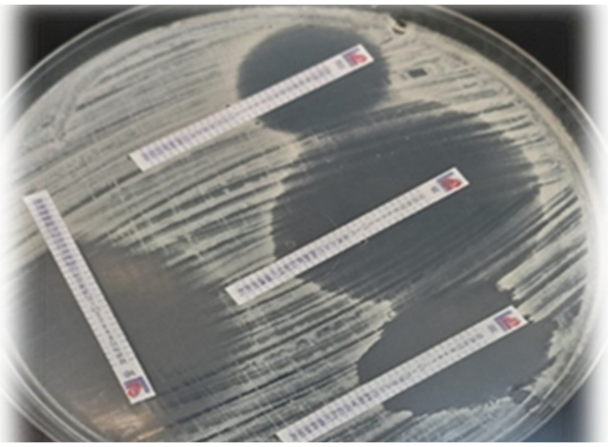

Figure S2. Antimicrobial suceptibility test. Mueller Hinton agar /w 5% dextrose containing Epsilometer test (E-Test) strips of voriconazole, itraconazole, fluconazole, and ketoconazole

Table S1: MIC values of 4 azole drugs against 79 clinical fungi isolates from pet dogs and cats

	Drug MIC range (μg/mL)			
**Fungal species**	Voriconazole 0.002-32ug/mL	Itraconazole 0.002-32ug/mL	Fluconazole 0.016-256ug/mL	Ketoconazole 0.002-32ug/mL
**Dermatophytes (*Trichophyton*)**				
**n=2**	2.5	1	0	1.5
M. pachydermatis				
**n=16**	0.047	0.125	4	0.032
**n=13**	0.047	0.125	6	0.032
**n=13**	0.064	0.125	6	0.032
**n=14**	0.064	0.19	8	0.064
C. tropicalis				
**n=2**	0.19	0.75	2	1
**n=2**	0.25	0.75	4	1
**n=4**	0.38	0.75	4	1.5
**n=2**	0.38	0.75	4	3
**n=4**	0.38	1.5	6	3
**n=2**	0.5	1.5	8	3
**n=2**	0.5	1.5	8	4
**n=1**	0.5	2	16	12
**n=2**	0.5	2	12	6

Note: MIC = minimal inhibitory concentration values for fungal species
